# Variation in *Pheidole nodus* (Hymenoptera: Formicidae) functional morphology across urban parks

**DOI:** 10.7717/peerj.15679

**Published:** 2023-07-18

**Authors:** Yi Luo, Qing-Ming Wei, Chris Newman, Xiang-Qin Huang, Xin-Yu Luo, Zhao-Min Zhou

**Affiliations:** 1 Key Laboratory of Southwest China Wildlife Resources Conservation (Ministry of Education), China West Normal University, Nanchong, China; 2 Nanchong Vocational and Technical College, Nanchong, China; 3 Wildlife Conservation Research Unit, Department of Biology, University of Oxford, Oxford, United Kingdom; 4 Key Laboratory of Environmental Science and Biodiversity Conservation (Sichuan Province), China West Normal University, Nanchong, China

**Keywords:** Functional morphology, Urban parks, Urbanization, Hypervolume, Population segregation

## Abstract

**Background:**

Habitat fragmentation and consequent population isolation in urban areas can impose significant selection pressures on individuals and species confined to urban islands, such as parks. Despite many comparative studies on the diversity and structure of ant community living in urban areas, studies on ants’ responses to these highly variable ecosystems are often based on assemblage composition and interspecific mean trait values, which ignore the potential for high intraspecific functional trait variation among individuals.

**Methods:**

Here, we examined differences in functional traits among populations of the generalist ant *Pheidole nodus* fragmented between urban parks. We used pitfall trapping, which is more random and objective than sampling colonies directly, despite a trade-off against sample size. We then tested whether trait-filtering could explain phenotypic differences among urban park ant populations, and whether ant populations in different parks exhibited different phenotypic optima, leading to positional shifts in anatomical morphospace through the regional ant meta-population.

**Results:**

Intraspecific morphological differentiation was evident across this urban region. Populations had different convex hull volumes, positioned differently over the morphospace.

**Conclusions:**

Fragmentation and habitat degradation reduced phenotypic diversity and, ultimately, changed the morphological optima of populations in this urban landscape. Considering ants’ broad taxonomic and functional diversity and their important role in ecosystems, further work over a variety of ant taxa is necessary to ascertain those varied morphological response pathways operating in response to population segregation in urban environments.

## Introduction

The paradigm of morphological shifting remains a central topic in evolutionary biology. Traditionally, ecogeographical rules (*e.g.*, Bergmann’s, Allen’s and Hesse’s rules) have often been used to explain spatial and/ or temporal patterns of morphological variation for endothermic (*e.g.*, Cui et al., 2020; Ryding et al., 2021) or ectothermic (*e.g.*, Arnan, Cerdá & Retana, 2015; Guilherme et al., 2019; Sosiak & Barden, 2021) species in response to gradients in environmental conditions, such as temperature. Variations in morphological traits may also result from phenotypic plasticity; that is, the capacity of a single genotype to produce a range of phenotypes under different environmental conditions (Gibbin et al., 2017); however, plasticity can be adaptive, non-adaptive or neutral depending on its relation to the optimal fitness in the changed environment (Ghalambor et al., 2007). Adaptive responses occur when the plastic response is favored by directional selection.

Environments may differ in their ecological optima (*i.e.,* a certain combination of ambient factors that is optimal for the growth, existence and reproduction of an organism), leading to directional selection and shifts in a population’s position within ecological space, or environments may differ in the range of ecological variation they can support (*i.e.,* in the strength of environmental filtering they impose), constraining the volume of ecological space occupied by a species, or population (Algar & López-Darias, 2016). These two options are not mutually exclusive and could act in concert or opposition. Morphological shifts can occur at different taxonomic levels, but typically involve the adaptation of populations to localized environmental conditions (Cohen, Haran & Dor, 2019; Cohen et al., 2021), especially when driven by ecological isolation.

Urbanization is an increasingly important driver of global change (Szulkin, Munshi-South & Charmantier, 2020; Des Roches et al., 2021). Urban ecological factors (*e.g.*, food availability, physiological demands, habitat modification, pollution, thermal landscape) create selective pressures that can have a strong effect on remnant populations, resulting in phenotypic traits that are divergent from conspecifics living in natural areas (*i.e.,* urban-derived phenotypes, Diamond & Martin, 2021; Diamond, Prileson & Martin, 2022). For example, in urban areas, neotropical lizard (*Anolis cristatellus*) populations exhibit longer limbs relative to body size, more sub-digital scales and a greater heat tolerance, although it remains to be established if this is genetically based (Winchell et al., 2016; Campbell-Staton et al., 2020). Importantly, phenotypic changes may occur within the lifespan of the individual (Cohen et al., 2021).

Within an urban ecosystem, isolated ‘green spaces’ represent ‘green islands’ with the potential to preserve faunistic and floristic assemblages. City parks often differ from one another in size, structure and complexity, and thus provide highly informative crucibles for studying how individual organisms adapt their tolerances, resulting in differentiation among populations enabling them to persist within them. Evidence of differentiation is scarce, but has been found both in invertebrates (De Carvalho et al., 2017) and vertebrates (Littleford-Colquhoun et al., 2017). Furthermore, Winchell et al. (2023) have shown that genomic parallelism could underlie phenotypic parallelism (*i.e.,* convergence), in response to consistent urban environmental trends.

Ants are an ecologically dominant faunal group in most terrestrial ecosystems, which play key ecological roles as nutrient cyclers, predators, soil engineers and regulators of plant growth and reproduction (Czechowski et al., 2012). Studies on ants have informed understanding on ecological responses to disturbance and land management (Andersen, 2019), and how they can provide a bioindicator of environmental degradation (Andersen, 1997; Andersen & Majer, 2004).

There is an extensive literature describing how ants adapt to urban park conditions; however, this mainly focuses on diversity and the structure of ant community assemblages (Clarke, Fisher & LeBuhn, 2008; Ślipiński, Zmihorski & Czechowski, 2012; Carpintero & Reyes-López, 2014; Reyes-López & Carpintero, 2014; Liu et al., 2019; Nooten et al., 2019). In contrast, there is a scarcity of research examining whether environmental heterogeneity among isolated urban parks can cause morphological shifts within fragmented ant populations. Given, however, that morphological shifts among worker ants from various species have been attributed to various environmental drivers in populations in natural habitats, morphological shifts adapting to urban habitats seem highly plausible (Heinze et al., 2003; Clémencet & Doums, 2007; Bernadou et al., 2016; Antonov, 2017; but see Gaudard, Robertson & Bishop, 2019). In these previous studies, homospecific populations tended to be characterized on the basis of the mean value of their functional traits, as though these represent a static unit in space and time. Ignoring intraspecific trait variation across paratypes could underestimate a species’ ability to compete with other community members (Ashton et al., 2010), the degree of niche and trait overlap (Courbaud, Vieilledent & Kunstler, 2012), resource use (Bolnick et al., 2003; Eggenberger et al., 2019) and potential responses to new conditions found in urban environments.

The prevalence of some ant generalist species in urban environments may be attributable to high functional morphology plasticity, enabling them to utilize different habitats. Examining workers of a local dominant ant species, *Pheidole nodus*, from urban parks in Nanchong city, China, here we tested for differences in how individuals from segregated populations fill morphological space (morphospace), and whether these differences arise due to the strength of environmental filtering (environmental filter-strength hypothesis). This would be implied if different phenotypic optima between park environments drive directional selection and shifts in a population’s position within morphospace. Alternatively, parks may differ in the range of ecological variation they can support (optimum-shift hypothesis), constraining the volume of ecological space occupied by each ant population. Either of these mechanisms in isolation, or combined, could potentially lead to shifts in the positions of populations in morphospace between habitats.

## Materials & Methods

### Study area

**Figure 1:**
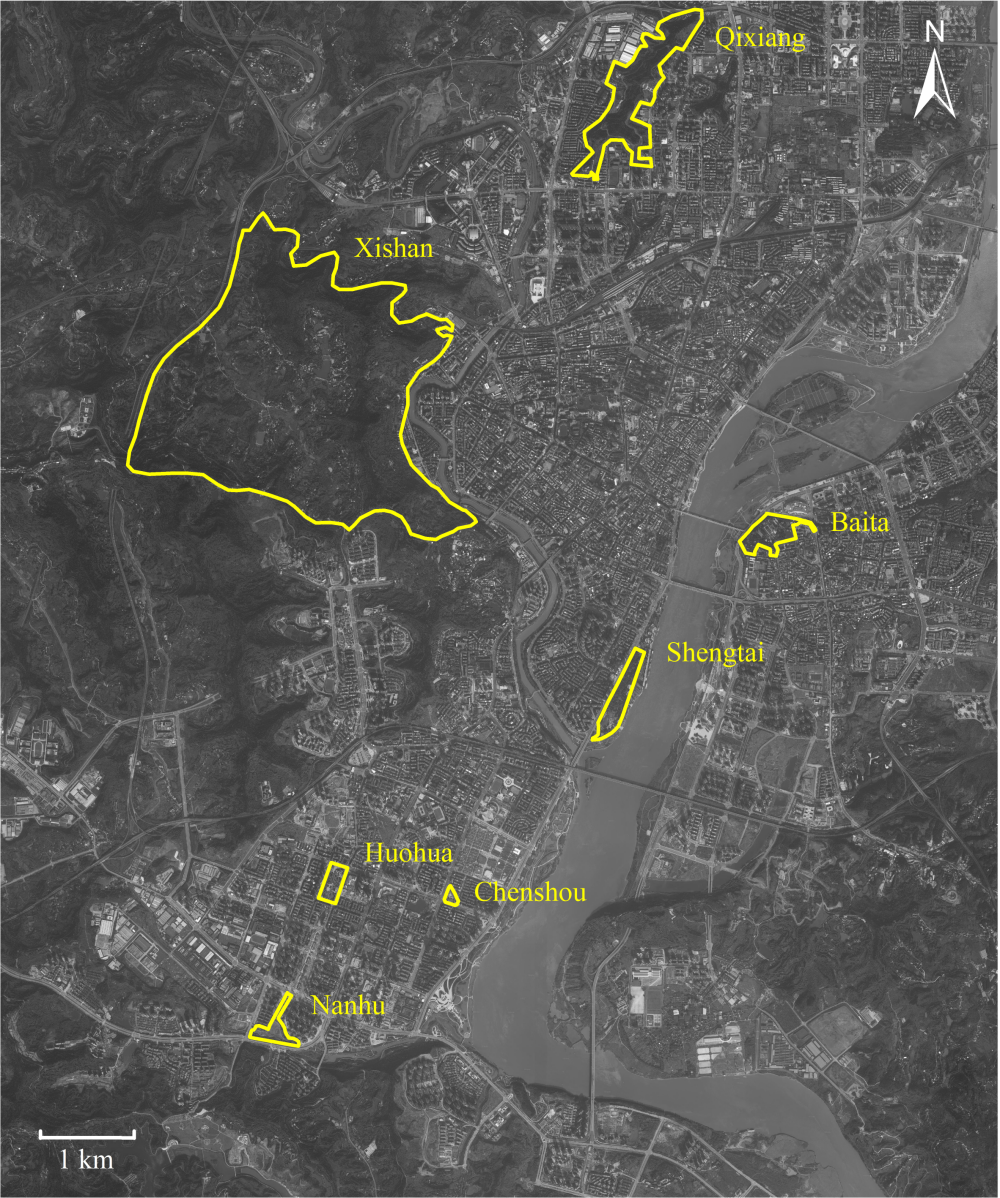
Map of Nanchong City showing the seven parks studied. Parks are highlighted with yellow borders. Map credit: Baidu Maps (https://map.baidu.com/).

This study was conducted in seven urban parks around downtown Nanchong (30°46′–31°85′N, 105°44′–106°96′E), located in Sichuan (China) (Fig. 1). This downtown area covers c. 160 square kilometers, occupied c. 1.5 million residents. The downtown core stretches north-south along the Jialing River. Nanchong has an East Asia monsoon climate, with an annual rainfall of 980 to 1,150 mm, a temperature range of 15.8 to 17.8 °C, and relative humidity from 76.0% to 86.0% (Liu et al., 2018; Luo et al., 2023).

### Ant sampling

*P. nodus* belongs to the subfamily Myrmicinae; a generalized functional group that copes well with disturbance (Hoffmann & Andersen, 2003; Kuate et al., 2015), dominating ant communities in warm, shady habitats where food resources are clumped and thus defensible (Andersen, 1997). We obtained foraging workers of *P. nodus* from seven urban parks (Fig. 1). We did not include workers that remain in the nest because our aim was to test the effects of environmental selection on ants actively foraging under urban habitat conditions. At six of these parks (Baita, 0.333 km^2^; Chenshou, 0.017 km^2^; Huohua, 0.067 km^2^; Nanhu, 0.373 km^2^; Qixiang, 0.700 km^2^; and Shengtai, 0.300 km^2^) the habitat was comprised mostly by impervious man-made surfaces, lawns and some sparse shrubs and trees planted deliberately (Fig. S1). In contrast, we also included Xishan Park; a larger park (18.8 km^2^) designated to protect the remnant native broad-leaved evergreen forest (Fig. S1). These seven parks were distributed 1.3 to 10.8 km apart across the high-density residential downtown district.

Ants were collected using pitfall traps from July to August 2020 during the summer season; the time of year when ant activity is greatest (Luo et al., 2023), maximizing foraging interactions with urban habitat conditions. At each park we established 16 pitfalls with at least 10 m spacing, to include all micro-habitat types and sample over as large an area as possible. Each trap consisted of a plastic container (7.1 cm diameter × 9 cm height) containing a solution of ethylene glycol (as a killing and preservative agent) and water at a 1:2 ratio. Each trap was covered by a small plastic plate attached to three nails to limit solution evaporation and rainfall ingress. Ants from each park were collected after sampling for 7 days and stored in 95% ethanol. We did not collect foraging workers and colony workers from nests because nests could not be located reliably due to only protruding c. five cm above ground and generally being concealed under shrubbery. Sampling ants predominantly from more obvious nests could potentially lead to sampling bias. Field experiments were approved by the Research Council of China West Normal University (project number: CWNU2020D002). Foraging workers of *P. nodus* were then identified based on taxonomic keys for ant fauna (Wu & Wang, 1995) and for the genus *Pheidole* (Pan, 2007). All specimens for this study were stored and examined at China West Normal University.

### Quantifying morphological characteristics

Morphological parameters were measured by Q.-M. Wei using a Leica M205C binocular microscope system and the ‘Leica Application Suite’ software. Six continuous morphological traits (Table 1) with functional significance were measured for each worker ant. These raw measurement data were used in a principal component analysis with a varimax rotation to identify key axes of morphological variation. The first four principal components that accounted for 88.16% of the total variance (PC1 = 49.46%, PC2 = 15.96%, PC3 = 12.37%, PC4 = 10.37%) were retained. PCs 1-4 were loaded heavily by traits related to body size, sensory abilities, prey size and capacity to explore habitat, respectively (Table 2, Fig. S2).

**Table 1:** Morphological traits, indicator of function and anatomical definition following Gibb et al. (2015) and Grevé et al. (2019).

Trait	Indicator of function	Measure
Weber’s length	Indicative of worker body size.	Distance from the anterodorsal margin of the pronotum to the posteroventral margin of the propodeum.
Head width	Size of gaps through which worker can pass, which is also a trait used as estimation of the worker size.	Maximum width of the head across the eyes.
Eye width	Indicative of the exploratory capacities of the habitat.	Measured across the maximum width of the eye.
Mandible length	Indicative of diet; longer mandibles could allow predation of larger prey.	Straight-line distance from the insertion to the tip of the mandible.
Scape length	Sensory abilities-longer scapes facilitate following of pheromone trails, finding food and perceiving vibrations, etc.	The maximum straight-line length of the scape, excluding the basal constriction.
Hindleg length	Leg length is the predictor for locomotory skills and habitat complexity.	Femur length and tibia length of one of the hindleg combined to leg length.

**Table 2:** Eigenvectors for the first four principal components from a principal components analysis of ant morphology after varimax rotation.

Variables	PC1	PC2	PC3	PC3
Head width	0.889	0.151	−0.029	0.161
Eye width	0.236	0.103	0.072	0.963
Mandible length	0.095	0.042	0.998	0.066
Scape length	0.305	0.938	0.041	0.104
Weber’s length	0.893	0.165	0.099	0.140
Hind leg length	0.690	0.299	0.172	0.164

### Testing the environmental filter-strength and optimum-shift hypotheses

We applied four PCs to analyze morphospace. In order to account for unequal sampling across parks, we randomly sub-sampled 10 workers from each park and then computed convex hulls with the ‘hypervolume’ package (Blonder et al., 2014; Blonder, 2015) in R ver. 4.1.1 (R Core Team, 2021). We repeated this sub-sampling 100 times; that is, we constructed 100 convex hulls for workers collected from each park. To assess significance, we generated a null expectation by randomly selecting 10 individuals from all the individuals collected, without replacement, and repeated this 1,000 times. Given that the null expectation exhibited a non-normal distribution, independent-sample Mann–Whitney *U* tests were employed to assess differences in convex hull volume and morphospace centroid (*i.e.,* the distance of a population’s centroid in morphospace from the overall centroid). In all analyses, significance was set at *P* < 0.001.

We also measured morphological dissimilarity (MD), which describes the percentage of total meta-population morphospace volume occupied uniquely by *P. nodus* workers from each population. To do so, we compared the MD of worker ants collected from separate parks, to include all 21 pairwise combinations, with the total MD among all parks.

To test the environmental filter-strength hypotheses, the convex hull volume for workers from each park was compared to the volume expected from a null model.

To test the optimum-shift hypothesis, the distance from each population’s morphospace centroid to the centroid of all populations pooled was calculated, and compared to a null expectation.

## Results

A total of 163 *P. nodus* workers were collected and measured; 19 from Baita, 15 from Chenshou, 15 from Huohua, 18 from Nanhu, 38 from Qixiang, 15 from Shengtai, and 43 from Xishan. Workers from a single park uniquely occupied 79.7% of total population morphospace dissimilarity (MD) (Table 3), which was significant based on our null model. The contribution of each individual park to MD ranged from 0.9 to 28.6%, where unique morphospace volume differed significantly from the null exception for each park except for Baita (Table 3). Among park pairs, MD ranged from 89.6 to 99.5%, and only six park population pairs (of 21; Chenshou-Qixiang, Chenchou-Xishan, Huohua-Qixiang, Huohua-Xishan, Qixiang-Shengtai and Shengtai-Xishan) occupied similar convex hull volumes (Table 4, Fig. S3).

**Table 3:** The contribution of individual parks to total morphological dissimilarity, *i.e.* the percentage of total morphospace volume uniquely occupied by ants from each park.

Park	Unique Volume (%)	*P*-value
Baita	20.1	=0.048
Chenshou	0.9	<0.001
Huohua	2.5	<0.001
Nanhu	28.6	<0.001
Qixiang	11.8	<0.001
Shengtai	2.7	<0.001
Xishan	13.1	<0.001

**Table 4:** Morphological turnover (% non-overlap, below diagonal) and P-values for the difference in convex hull volume (above the diagonal) among pairs of parks.

	Baita	Chenshou	Huohua	Nanhu	Qixiang	Shengtai	Xishan
Baita		<0.001	<0.001	<0.001	<0.001	<0.001	<0.001
Chenshou	99.5	–	<0.001	<0.001	0.299	<0.001	0.067
Huohua	95.5	97.6	–	<0.001	0.858	<0.001	0.016
Nanhu	97.8	93.6	97.2	–	<0.001	<0.001	<0.001
Qixiang	92.4	98.9	97.9	97.9	–	0.038	<0.001
Shengtai	97.3	86.6	95.7	90.9	97.5	–	0.003
Xishan	91.9	95.4	93.4	93.3	96.0	89.6	–

**Figure 2:**
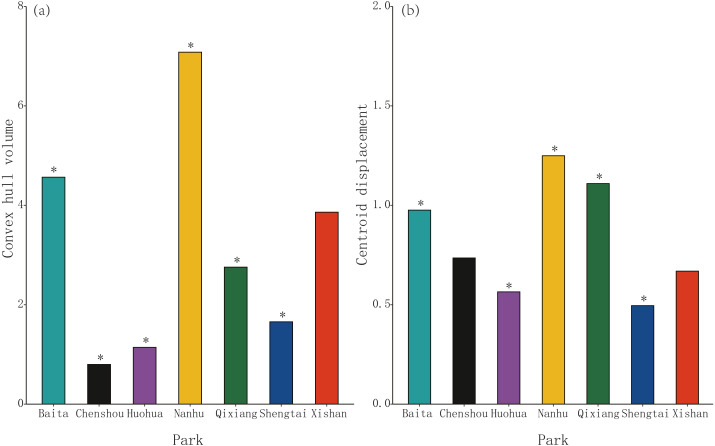
The volume and position of morphospace occupied by sub-populations of *Pheidole nodus* collected from the seven parks in Nanchong City (Fig. 1). Convex hull volume (A) indicates the extent of trait-clustering, while centroid displacement (B) depicts the distance of each sub-population’s morphospace centroid from the pooled centroid across all populations. An asterisk (*) indicates a *P*-value less than 0.001 based on the null model.

Morphospace (convex hull) volume clustering was significant at four of these seven parks, Chenshou (median value = 0.80), Huohua (1.14), Qixiang (2.76) and Shengtai (1.66), but was not significant for Xishan (3.86, *P* = 0.975); significantly larger convex hull volumes were evident at Baita (4.57) and Nanhu (8.50) parks. (Fig. 2A). Centroid displacement was significantly greater than the null expectation value at Baita (median population distance from pooled centroid across all populations = 0.98), Nanhu (1.25) and Qixiang (1.11) parks, but was not significant for Chenshou (0.74, *P* = 0.100) and Xishan (0.67, *P* = 0.624); significantly lower centroid displacements were evident for Huohua (0.57) and Shengtai (0.50) (Fig. 2B).

## Discussion

We found that *P. nodus* populations from urban parks spaced several kilometers apart exhibited distinct morphometric traits variations. Although these seven urban populations were likely entirely segregated from each other, morphospace volumes at four of the seven urban parks were smaller than the null expectation, and centroid displacements at three of these urban parks exceeded the null expectation, suggesting trait clustering and a shift in trait optima. These results are highly consistent with the environmental filter-strength hypothesis, which proposes that the strength of environmental filtering varies across environments, leading to differential trait-clustering (phenotypic variance). Simultaneously, these results also support the optimum-shift hypothesis, which proposes that the phenotypic optimum differs between environments, leading to divergence in phenotypic morphospace position between populations in different environments (see: Algar & López-Darias, 2016). From this we deduce that generalist ant species, such as *P. nodus*, can exhibit both substantial intraspecific variation in phenotypic diversity and optima in response to differences in habitat conditions.

Furthermore, we also found substantial segregation for both phenotypic clustering and optima between populations. This suggests that ant inter-population dispersal *via* spillover between these urban parks was severely limited, due to habitat fragmentation. This is typical in urban landscapes, where residential, commercial and industrial development, along with roads and paved pedestrian areas present effective dispersal barriers, especially for species with restricted dispersal distances (Fletcher Jr, Reichert & Holmes, 2018). Indeed, the urban landscape habitat matrix presents a generally inhospitable and ubiquitous barrier to colonization by many urban-avoiding terrestrial arthropods (Ricketts, 2001; Schmidt, 2008; Brühl & Eltz, 2010). Recent studies of ants conducted across Manhattan’s urban habitat mosaic (Savage et al., 2015), as well as in the suburban riparian corridor in Ku-ring-gai Local Government Area, Sydney, Australia (Ives et al., 2011) and across urban parks in Taichung, China (Liu et al., 2019) all found similar evidence of impeded spillover between fragmented populations. As for *P. nodus* specifically, its dispersal potential is unknown but, in general, ants are poor dispersers, with genetically viscous population structures (Sundström, Seppä & Pamilo, 2005; Hakala, Perttu & Helanterä, 2019).

Land-use disturbance arising due to urbanization generally intensifies selective pressures that reduce diversity and causes biotic homogenization (Rocha-Ortega & Castaño Meneses, 2015; Morelli et al., 2016). Consequently, locally invasive non-native biotas often steadily replace native biotas. Our finding that ant populations in Chenshou, Huohua, Qixiang and Shengtai parks had more homogeneous morphological trait patterns suggests that selective pressures in these urban sites favor reduced phenotypic plasticity in corroboration of this homogenization scenario. Similar intra-specific canalization of phenotypic diversity has been noted for other taxa in urban settings, such as mosquitos (*Culex nigripalpus*) (De Carvalho et al., 2017), Puerto Rican Crested Anoles lizards (*Anolis cristatellus*) (Winchell et al., 2016; Winchell et al., 2018) and neotropical Wedge-billed Woodcreepers (*Glyphorynchus spirurus*) (Thompson et al., 2022). In contrast, urban conditions appear to drive phenotypic diversity in bumblebees (*Bombus pascuorum* and *Bombus lapidarius*), because urban landscaping and management can lead to cities having a greater morphological variety of flowering species in any one season compared to rural areas (Eggenberger et al., 2019).

In our study, the population at Xishan Park was exceptional, where neither morphospace volume nor centroid displacement were distinct from that expected based on random sampling across all the collected individuals. Plausibly, this may be because this park is much larger than the others we sampled and continues to support a remnant of more natural native broad-leaved evergreen forest, providing habitat diversity and a broader range of food types. That the intra-specific morphological diversity among *P. nodus* workers under Xishan Park’s more natural conditions was around average, compared to the full meta-population sampled, suggests that these ants have evolved an optimal base morphology suited to their most natural habitat.

Mechanistically, it remains difficult at this stage to resolve whether these functional trait shifts are (i) an adaptive response to urban conditions, (ii) simply an example of background capacity for phenotypic plasticity, or (iii) based on genetics or epigenetics. Although *P. nodus* belongs to the generalized Myrmicinae functional group, which prefer open habitats and are often favored by disturbance (Andersen, 2019), the tendency for phenotypic clustering and optimum displacement in urban populations suggests that this species does not exhibit pre-adaptations (exaptation; *sensu* Gould & Vrba, 1982) to urban conditions, unlike (for example) from cockroaches, pigeons and rats. Plausibly phenotypic characteristics differ among urban populations due to myriad novel environmental effects on ontological development. To test for a heritable genetic component would require testing the fitness of F1 and F2 offspring reared in a controlled environment (McDonnell & Hahs, 2015). Alternatively, if workers inherit traits from the queen that contribute to colony fitness, this would provide an adaptive explanation. Any heritable genotypic adaptation to novel environmental conditions would arise through either of two selective mechanisms: (1) the expression of phenotypic plasticity (the ability of one genotype to express varying phenotypes when exposed to different environmental conditions); and (2) evolution *via* directed selection for particular phenotypes, resulting in the modification of the population gene pool (Clusella-Trullas, Terblanche & Chown, 2010; Fox et al., 2019; Desmond, 2021; Jacquier et al., 2021; Lambert et al., 2021). Even in the absence of genetic data, the reduced morphospace and centroid displacement we detected are consistent with urban habitat fragmentation reducing genetic diversity within urban populations, while simultaneously increasing populations genetic differentiation from a standard neutral model. Such neutral processes are typically associated with detrimental fitness consequences, in part arising from fragmentation and population isolation of that leads to increased drift and elevated probability of inbreeding depression (Combs et al., 2018).

Ours is the first preliminary study to report differences in variance of ant functional morphology at species level among urban parks. Our study involved a relatively small sample size, due to the low abundance of ants in this highly disturbed region collected using a pitfall sampling method, chosen to sample morphological traits from only foraging workers experiencing urban habitat conditions, and to exclude colony workers. On the basis of this sample size, we caution that those differences in the morphospaces of each park population we detected, while indicative, should not be considered conclusive until further work builds on this foundation to more fully quantify the effects of ecological filters on intraspecific morphological variation among ants in urban micro-habitats.

## Conclusions

The evidence we found for morphological distinctiveness and segregation for both phenotypic clustering and optima between populations implies that filtering and selective mechanisms may both be operating on ant functional morphological traits. Ultimately, however, it remains to be seen whether *P. nodus* will have sufficient plasticity to continue to persist faced with the novel challenges of urban habitats, especially with concomitant climate change pressures (Goh, 2020), and will be robust to those Allee effects that can perturb isolated populations (Schoereder et al., 2004). Nevertheless, our results begin to demonstrate how novel urban environments present challenges to ant generalist populations, to which species respond through phenotypic, and possibly adaptive, change.

##  Supplemental Information

10.7717/peerj.15679/supp-1 Figure S1 Sampling habitats at Baita (a), Chenshou (b), Huohua (c), Nanhu (d), Qixiang (e) Shengtai (f) and Xishan (g) parks in Nanchong City, China

10.7717/peerj.15679/supp-2 Figure S2 Plots on PCs for functional measurements of *Pheidole nodus* from Baita, Chenshou, Huohua, Nanhu, Qixiang, Shengtai and Xishan parks in Nanchong City, China

10.7717/peerj.15679/supp-3 Figure S3 The convex hulls of *Pheidole nodus* workers collected from seven parks in Nanchong city (Fig. 1), based on PCs (PC1 loaded heavily on body size, PC2 on sensory abilities, PC3 on prey size and PC4 on exploratory capacities of the habitat)

10.7717/peerj.15679/supp-4 Data S1 Measurements of ant individuals from seven urban parks The raw data shows the functional morphology of all ant individuals which were collected from urban parks. These ants were used for statistical analysis to compare populations among parks.

## Additional Information and Declarations

### Field Study Permissions

The following information was supplied relating to field study approvals (*i.e.*, approving body and any reference numbers): Field experiments were approved by the Research Council of China West Normal University (project number: CWNU2020D002)

### Data Availability

The following information was supplied regarding data availability: The raw measurements are available in the Supplemental Files.

## References

[ref-1] Algar AC, López-Darias M (2016). Sex-specific responses of phenotypic diversity to environmental variation. Ecography.

[ref-2] Andersen A (1997). Functional groups and patterns of organization in north American ant communities: a comparison with Australia. Journal of Biogeography.

[ref-3] Andersen AN (2019). Responses of ant communities to disturbance: five principles for understanding the disturbance dynamics of a globally dominant faunal group. Journal of Animal Ecology.

[ref-4] Andersen AN, Majer JD (2004). Ants show the way down under: invertebrates as bioindicators in land management. Frontiers in Ecology and the Environment.

[ref-5] Antonov IA (2017). Interpopulation variation in morphometric characteristics of the ant *Myrmica angulinodis Ruzs* (Hymenoptera: Formicidae) in the Baikal region. Russian Journal of Ecology.

[ref-6] Arnan X, Cerdá X, Retana J (2015). Partitioning the impact of environment and spatial structure on alpha and beta components of taxonomic, functional, and phylogenetic diversity in European ants. PeerJ.

[ref-7] Ashton IW, Miller AE, Bowman WD, Suding KN (2010). Niche complementarity due to plasticity in resource use: plant partitioning of chemical N forms. Ecology: A Publication of the Ecological Society of America.

[ref-8] Bernadou A, Roemermann C, Gratiashvili N, Heinze J (2016). Body size but not colony size increases with altitude in the holarctic ant, Leptothorax acervorum. Ecological Entomology.

[ref-9] Blonder B (2022). https://cran.r-project.org/web/packages/hypervolume/index.html.

[ref-10] Blonder B, Lamanna C, Violle C, Enquist BJ (2014). The n-dimensional hypervolume. Global Ecology and Biogeography.

[ref-11] Bolnick DI, Svanbäck R, Fordyce JA, Yang LH, Davis JM, Hulsey CD, Forister ML (2003). The ecology of individuals: incidence and implications of individual specialization. American Naturalist.

[ref-12] Brühl CA, Eltz T (2010). Fuelling the biodiversity crisis: species loss of ground-dwelling forest ants in oil palm plantations in Sabah, Malaysia (Borneo). Biodiversity and Conservation.

[ref-13] Campbell-Staton SC, Winchell KM, Rochette NC, Fredette J, Maayan I, Schweizer RM, Catchen J (2020). Parallel selection on thermal physiology facilitates repeated adaptation of city lizards to urban heat islands. Nature Ecology and Evolution.

[ref-14] Carpintero S, Reyes-López J (2014). Effect of park age, size, shape and isolation on ant assemblages in two cities of southern Spain. Entomological Science.

[ref-15] Clarke KM, Fisher BL, LeBuhn G (2008). The influence of urban park characteristics on ant (Hymenoptera, Formicidae) communities. Urban Ecosystems.

[ref-16] Clémencet J, Doums C (2007). Habitat-related microgeographic variation of worker size and colony size in the ant *Cataglyphis cursor*. Oecologia.

[ref-17] Clusella-Trullas S, Terblanche JS, Chown SL (2010). Phenotypic plasticity of locomotion performance in the seed harvester *Messor capensis* (Formicidae). Physiological and Biochemical Zoology.

[ref-18] Cohen TM, Haran R, Dor R (2019). Signals of local adaptation across an environmental gradient among highly connected populations of the Dead Sea sparrow *Passer moabiticus* in Israel. Ibis.

[ref-19] Cohen TM, Major RE, Kumar RS, Nair M, Ewart KM, Hauber ME, Dor R (2021). Rapid morphological changes as agents of adaptation in introduced populations of the common myna (*Acridotheres tristis*). Evolutionary Ecology.

[ref-20] Combs M, Byers KA, Ghersi BM, Blum MJ, Caccone A, Costa F, Himsworth CG, Richardson JL, Munshi-South J (2018). Urban rat races: spatial population genomics of brown rats (*Rattus norvegicus*) compared across multiple cities. Proceedings of the Royal Society B Biological Sciences.

[ref-21] Courbaud B, Vieilledent G, Kunstler G (2012). Intra-specific variability and the competition-colonisation trade-off: coexistence, abundance and stability patterns. Theoretical Ecology.

[ref-22] Cui J, Lei B, Newman C, Ji S, Su H, Buesching CD, Macdonald DW, Zhou Y (2020). Functional adaptation rather than ecogeographical rules determine body-size metrics along a thermal cline with elevation in the Chinese pygmy dormouse (*Typhlomys cinereus*). Journal of Thermal Biology.

[ref-23] Czechowski W, Radchenko A, Czechowska W, Vepsäläinen K (2012). The ants of Poland with reference to the myrmecofauna of Europe.

[ref-24] De Carvalho GC, Vendrami DP, Marrelli MT, Wilke A (2017). Wing variation in *Culex nigripalpus* (Diptera: Culicidae) in urban parks. Parasite Vector.

[ref-25] Des Roches S, Brans KI, Lambert MR, Rivkin LR, Savage AM, Schell CJ, Correa C, Meester LD, Diamond SE, Grimm NB, Harris NC, Govaert L, Hendry AP, Johnson MTJ, Munshi-South J, Palkovacs EP, Szulkin M, Urban MC, Verrelli BC, Alberti M (2021). Socio-eco-evolutionary dynamics in cities. Evolutionary Applications.

[ref-26] Desmond H (2021). Adapting to environmental heterogeneity: selection and radiation. Biological Theory.

[ref-27] Diamond SE, Martin RA (2021). Evolution in cities. Annual Review of Ecology, Evolution, and Systematics.

[ref-28] Diamond SE, Prileson E, Martin RA (2022). Adaptation to urban environments. Current Opinion in Insect Science.

[ref-29] Eggenberger H, Frey D, Pellissier L, Ghazoul J, Fontana S, Moretti M (2019). Urban bumblebees are smaller and more phenotypically diverse than their rural counterparts. Journal of Animal Ecology.

[ref-30] Fletcher Jr RJ, Reichert BE, Holmes K (2018). The negative effects of habitat fragmentation operate at the scale of dispersal. Ecology.

[ref-31] Fox RJ, Donelson JM, Schunter C, Ravasi T, Gaitán-Espitia JD (2019). Beyond buying time: the role of plasticity in phenotypic adaptation to rapid environmental change. Proceedings of the Royal Society B Biological Sciences.

[ref-32] Gaudard CA, Robertson MP, Bishop TR (2019). Low levels of intraspecific trait variation in a keystone invertebrate group. Oecologia.

[ref-33] Ghalambor CK, McKay JK, Carroll SP, Reznick DN (2007). Adaptive versus non-adaptive phenotypic plasticity and the potential for contemporary adaptation in new environments. Functional Ecology.

[ref-34] Gibb H, Stoklosa J, Warton DI, Brown AM, Andrew NR, Cunningham SA (2015). Does morphology predict trophic position and habitat use of ant species and assemblages?. Oecologia.

[ref-35] Gibbin EM, Massamba N’Siala G, Chakravarti LJ, Jarrold MD, Calosi P (2017). The evolution of phenotypic plasticity under global change. Scientific Reports.

[ref-36] Goh K (2020). Flows in formation: the global-urban networks of climate change adaptation. Urban Studies.

[ref-37] Gould SJ, Vrba ES (1982). Exaptation—a missing term in the science of form. Paleobiology.

[ref-38] Grevé ME, Bláha S, Teuber J, Rothmaier M, Feldhaar H (2019). The effect of ground surface rugosity on ant running speed is species-specific rather than size dependent. Insectes Sociaux.

[ref-39] Guilherme DR, Souza JLP, Franklin E, Pequeno PACL, Das Chagas AC, Baccaro FB (2019). Can environmental complexity predict functional trait composition of ground-dwelling ant assemblages? A test across the Amazon Basin. Acta Oecologica.

[ref-40] Hakala SM, Perttu S, Helanterä H (2019). Evolution of dispersal in ants (Hymenoptera: Formicidae): a review on the dispersal strategies of sessile superorganisms. Myrmecological News.

[ref-41] Heinze J, Foitzik S, Fischer B, Wanke T, Kipyatkov VE (2003). The significance of latitudinal variation in body size in a holarctic ant, Leptothorax acervorum. Ecography.

[ref-42] Hoffmann BD, Andersen AN (2003). Responses of ants to disturbance in Australia, with particular reference to functional groups. Austral Ecology.

[ref-43] Ives CD, Hose GC, Nipperess DA, Taylor MP (2011). Environmental and landscape factors influencing ant and plant diversity in suburban riparian corridors. Landscape and Urban Planning.

[ref-44] Jacquier L, Doums C, Four-Chaboussant A, Peronnet R, Tirard C, Molet M (2021). Urban colonies are more resistant to a trace metal than their forest counterparts in the ant *Temnothorax nylanderi*. Urban Ecosystems.

[ref-45] Kuate AF, Hanna R, Tindo M, Nanga S, Nagel P (2015). Ant diversity in dominant vegetation types of southern Cameroon. Biotropica.

[ref-46] Lambert MR, Brans KI, Des Roches S, Donihue CM, Diamond SE (2021). Adaptive evolution in cities: progress and misconceptions. Trends in Ecology & Evolution.

[ref-47] Littleford-Colquhoun BL, Clemente C, Whiting MJ, Ortiz-Barrientos D, Frère CH (2017). Archipelagos of the Anthropocene: rapid and extensive differentiation of native terrestrial vertebrates in a single metropolis. Molecular Ecology.

[ref-48] Liu KL, Peng MH, Hung YC, Neoh KB (2019). Effects of park size, peri-urban forest spillover, and environmental filtering on diversity, structure, and morphology of ant assemblages in urban park. Urban Ecosystems.

[ref-49] Liu S, Yu WH, Li F, Zhao J, Yin RY, Zhou ZM, Pan B (2018). Fertilizer application in rural cropland drives cadmium enrichment in bats dwelling in an urban area. Environmental Pollution.

[ref-50] Luo XY, Newman C, Luo Y, Zhou ZM (2023). Comparing ant assemblages and functional groups across urban habitats and seasons in an east asia monsoon climate area. Animals.

[ref-51] McDonnell MJ, Hahs AK (2015). Adaptation and adaptedness of organisms to urban environments. Annual Review of Ecology, Evolution, and Systematics.

[ref-52] Morelli F, Benedetti Y, Ibáñez Álamo JD, Jokimäki J, Mänd R, Tryjanowski P, Møller AP (2016). Evidence of evolutionary homogenization of bird communities in urban environments across Europe. Global Ecology and Biogeography.

[ref-53] Nooten S, Schultheiss P, Rowe RC, Facey SL, Cook JM (2019). Habitat complexity affects functional traits and diversity of ant assemblages in urban green spaces (Hymenoptera: Formicidae). Myrmecological News.

[ref-54] Pan Y (2007). Systematic study on the ant genera *Pheidole* Westwood and *Aphaenogaster Mayr* (Hymenoptera: Formincidae: Myrmicinae) in China. Thesis.

[ref-55] R Core Team (2021).

[ref-56] Reyes-López J, Carpintero S (2014). Comparison of the exotic and native ant communities (Hymenoptera: Formicidae) in urban green areas at inland, coastal and insular sites in Spain. European Journal of Entomology.

[ref-57] Ricketts TH (2001). The matrix matters: effective isolation in fragmented landscapes. The American Naturalist.

[ref-58] Rocha-Ortega M, Castaño Meneses G (2015). Effects of urbanization on the diversity of ant assemblages in tropical dry forests, Mexico. Urban Ecosystems.

[ref-59] Ryding S, Klaassen M, Tattersall GJ, Gardner JL, Symonds MRE (2021). Shape-shifting: changing animal morphologies as a response to climatic warming. Trends in Ecology & Evolution.

[ref-60] Savage AM, Hackett B, Guénard B, Youngsteadt EK, Dunn RR (2015). Fine-scale heterogeneity across Manhattan’s urban habitat mosaic is associated with variation in ant composition and richness. Insect Conservation and Diversity.

[ref-61] Schmidt C (2008). Phylogeny of the terrestrial Isopoda (Oniscidea): a review. Arthropod Systematics and Phylogeny.

[ref-62] Schoereder JH, Sobrinho TG, Ribas CR, Campos RBF (2004). Colonization and extinction of ant communities in a fragmented landscape. Austral Ecology.

[ref-63] Ślipiński P, Zmihorski M, Czechowski W (2012). Species diversity and nestedness of ant assemblages in an urban environment. European Journal Entomology.

[ref-64] Sosiak CE, Barden P (2021). Multidimensional trait morphology predicts ecology across ant lineages. Functional Ecology.

[ref-65] Sundström L, Seppä P, Pamilo P (2005). Genetic population structure and dispersal patterns in Formica ants—a review. Annales zoologici fennici.

[ref-66] Szulkin M, Munshi-South J, Charmantier A (2020). Urban evolutionary biology.

[ref-67] Thompson MJ, Capilla-Lasheras P, Dominoni DM, Réale D, Charmantier A (2022). Phenotypic variation in urban environments: mechanisms and implications. Trends in Ecology & Evolution.

[ref-68] Winchell KM, Campbell-Staton SC, Losos JB, Revell LJ, Verrelli BC, Geneva AJ (2023). Genome-wide parallelism underlies contemporary adaptation in urban lizards. Proceedings of the National Academy of Sciences of the United States of America.

[ref-69] Winchell KM, Maayan I, Fredette JR, Revell LJ (2018). Linking locomotor performance to morphological shifts in urban lizards. Proceedings of Royal Society B Biological Sciences.

[ref-70] Winchell KM, Reynolds RG, Prado-Irwin SR, Puente-Rolón AR, Revell LJ (2016). Phenotypic shifts in urban areas in the tropical lizard *Anolis cristatellus*. Evolution.

[ref-71] Wu J, Wang C (1995). The ants of China.

