# Supplementary figures and images for: Variation in Pheidole nodus (Hymenoptera: Formicidae) functional morphology across urban parks
Source: PeerJ. 2023 Jul 18;11:e15679. doi: 10.7717/peerj.15679 (PMC10361077; doi:10.7717/peerj.15679)

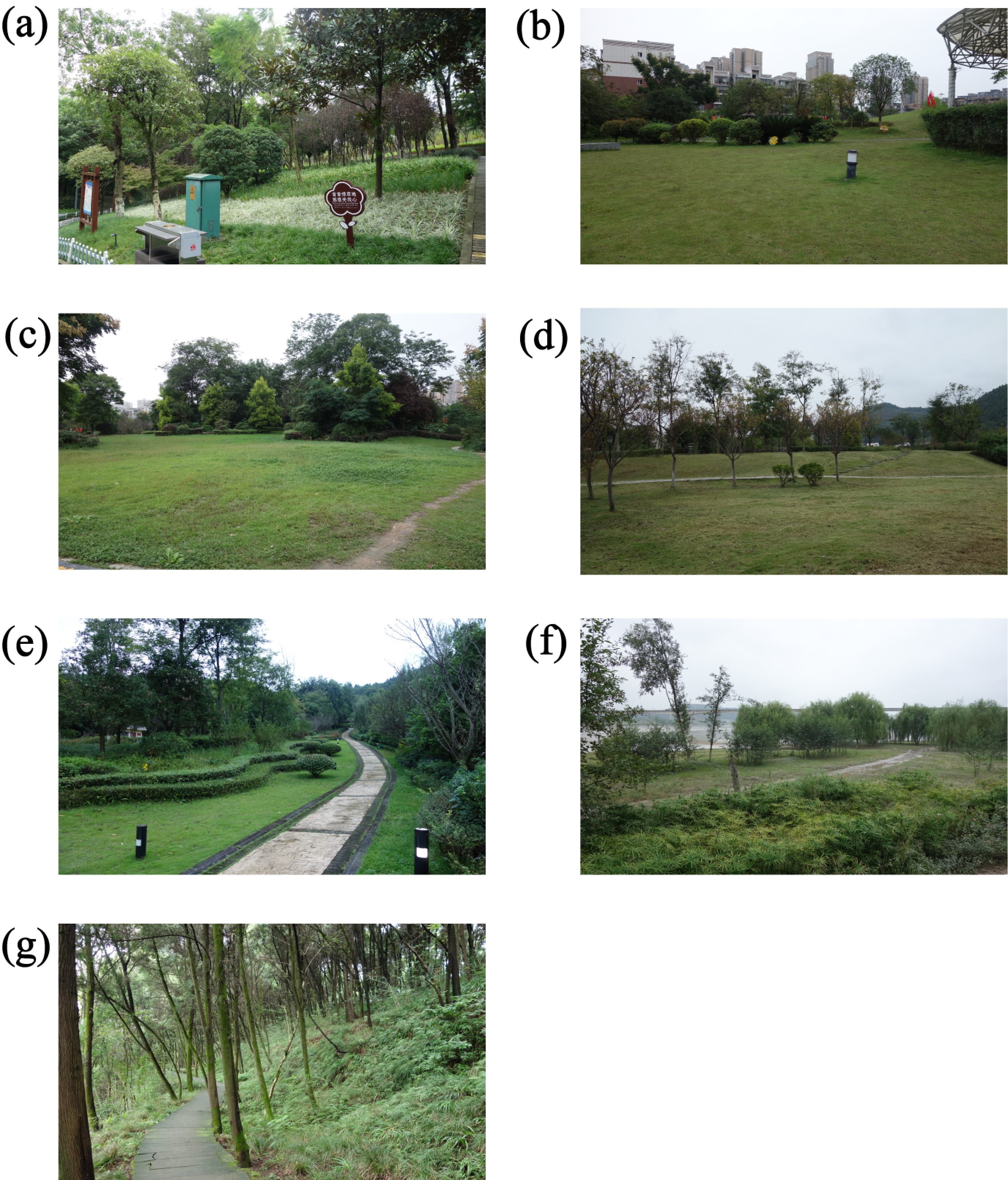

Supplement: Figure S1 [file peerj-11-15679-s001.png]

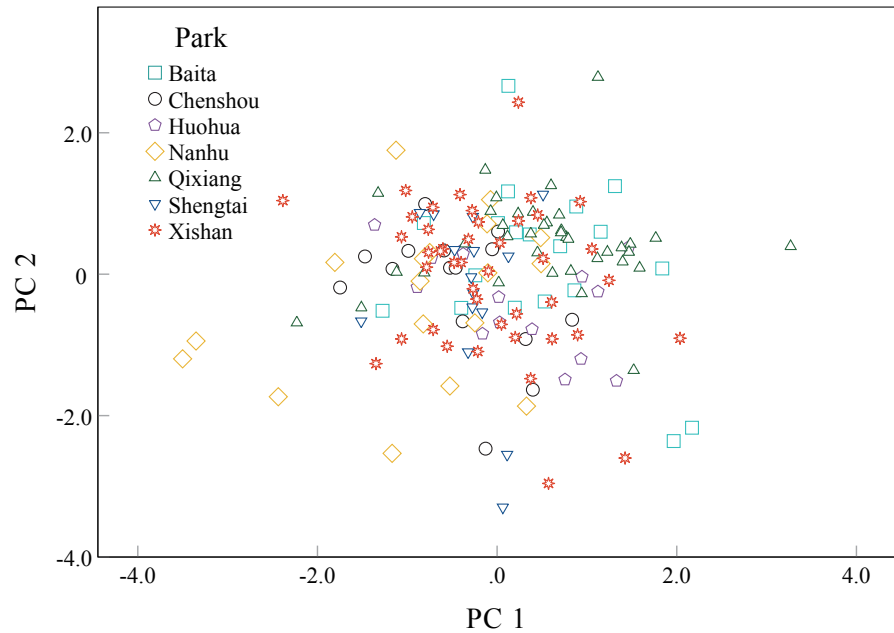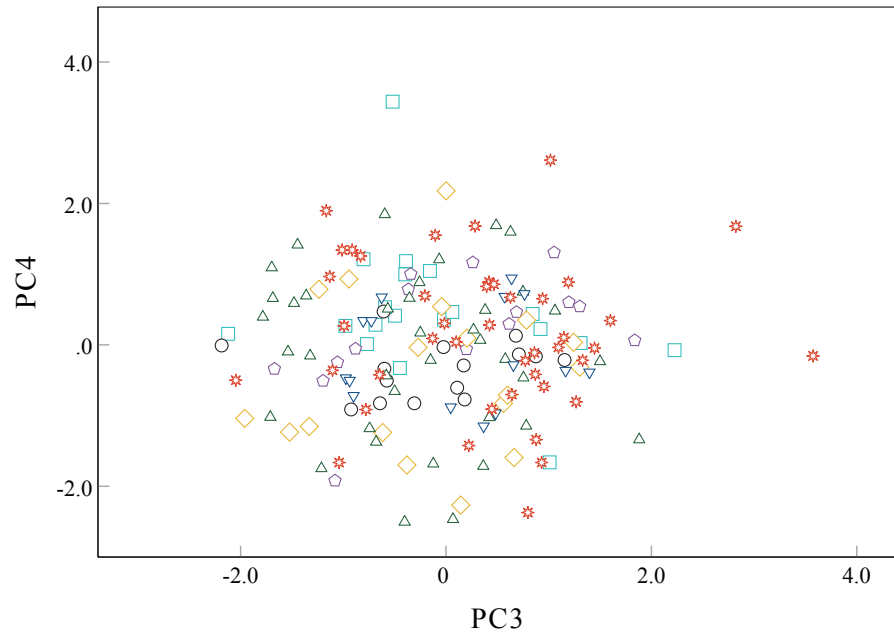

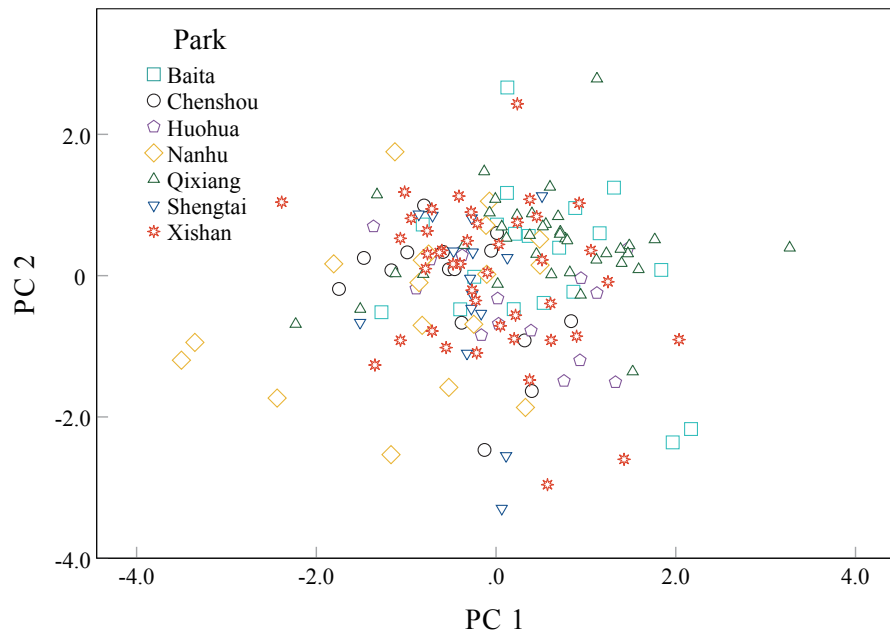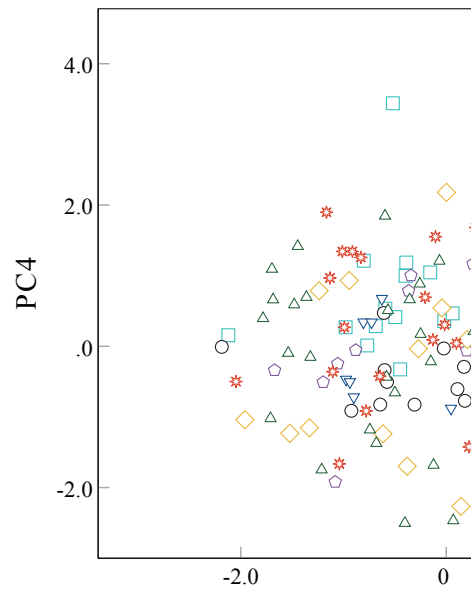

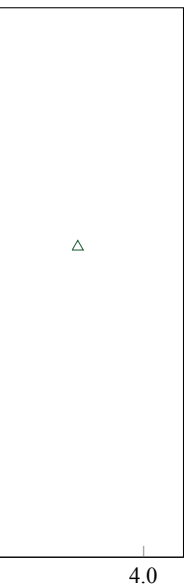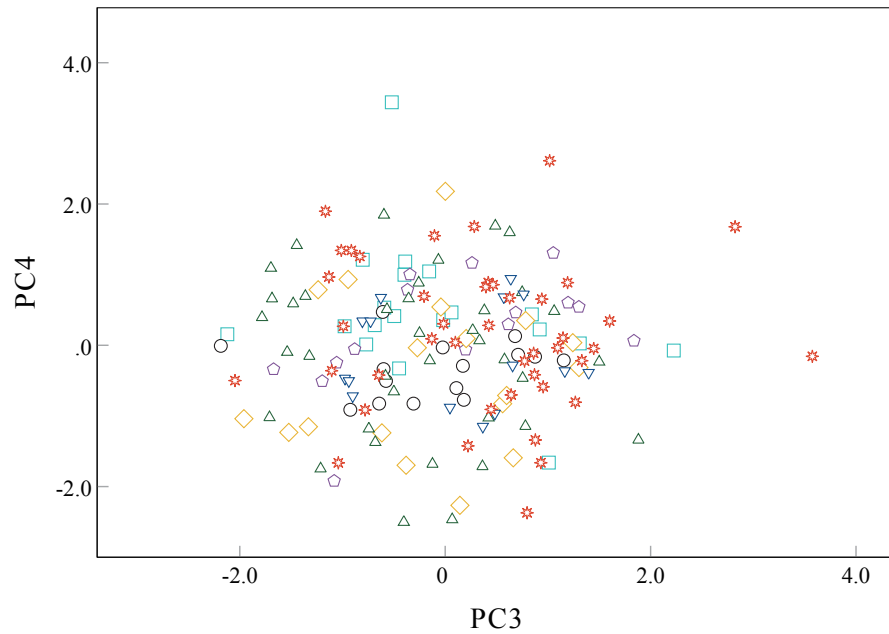

Supplement: Figure S2 [file peerj-11-15679-s002.pdf]

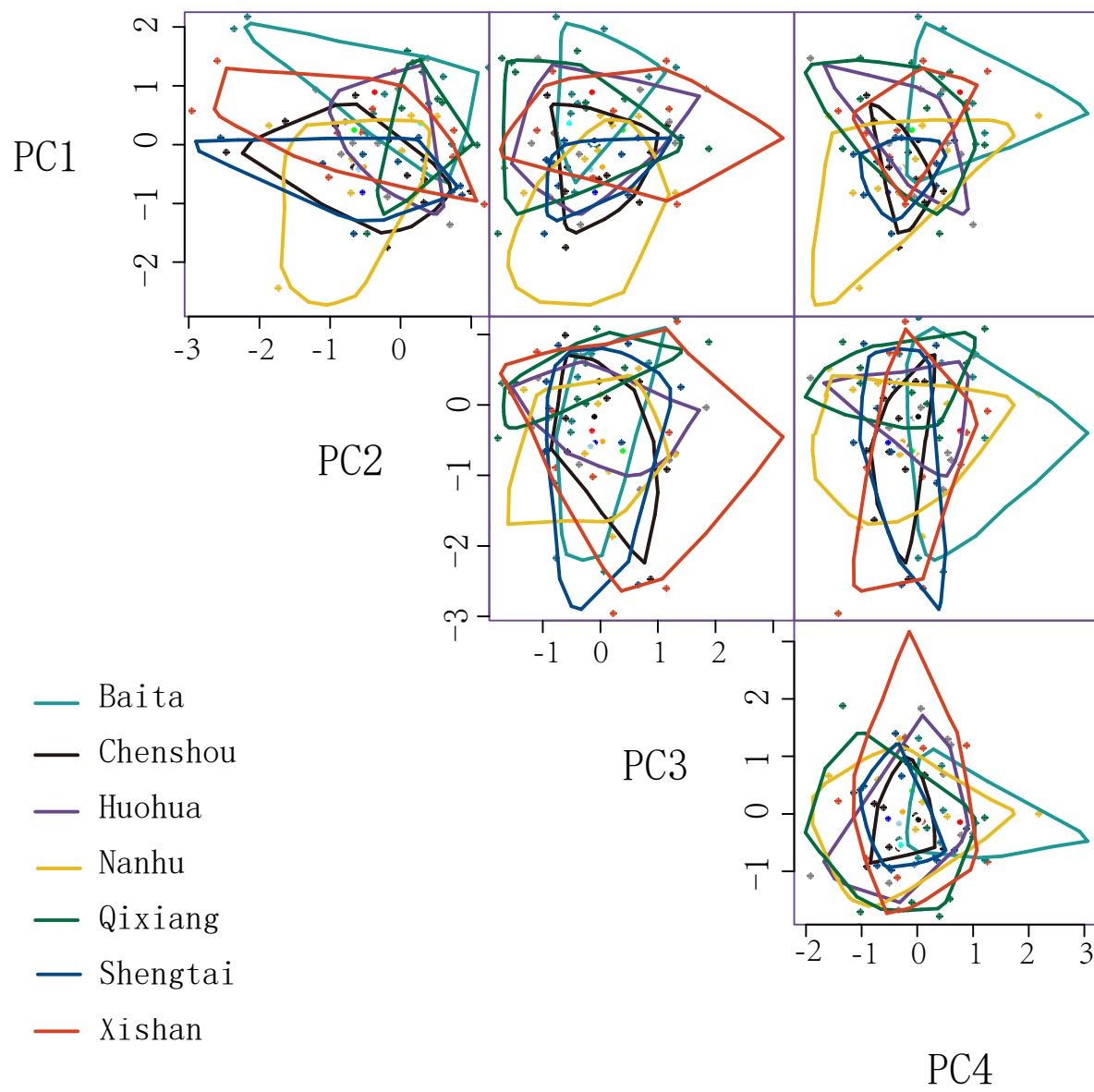

Supplement: Figure S3 [file peerj-11-15679-s003.pdf]
